# Anchorage performance of a new rebar bolt under different surrounding rock strength and borehole depth

**DOI:** 10.1038/s41598-024-59771-4

**Published:** 2024-04-26

**Authors:** Ming Zhang, Chen Cao, Guanghan Li, Baolong Guo

**Affiliations:** 1 Research Center of Emergency Management Department, Beijing, People’s Republic of China; 2 College of Mining, Liaoning Technical University, Fuxin, People’s Republic of China

**Keywords:** Engineering, Civil engineering

## Abstract

The theory and technology of rock bolting are fundamental research topics for strata control in civil and mining engineering. Rebar bolts are commonly used for roadway primary support in underground coal mine. To adapt to deep resource mining, a new left threaded rebar bolt has been developed. Compared to conventional rebar bolts, the result of installation test showed that the new bolt reduced of 41.5% and 57.9% in stirring resistance force and torque, respectively. In laboratory pullout tests, PVC and aluminum sleeves were used to simulate weak and medium strength surrounding rocks. The average peak pullout force, displacement at the peak load and energy absorption increased by 27%, 107% and 108%, respectively, using PVC sleeve; and increased by 113%, 109% and 212%, respectively, using aluminum sleeve. Field tests were conducted under soft coal, hard coal and medium strength rock geo-conditions. Different borehole depths were selected to precisely calculate the average anchorage performance of the new bolt. Results showed that the average peak pullout force of the new bolt increased by 37%, 38% and 28%, respectively, under different surrounding rock conditions. Moreover, based on on-site test results, the pullout curves in field-testing were summarised and classified into 6 different patterns, which were discussed from a viewpoint of causality mechanism. The research findings validate that the newly developed bolt has better anchorage performance compared to conventional rebar bolts, making it a new anchorage material for deep resource mining.

## Introduction

To meet the growing energy demands, the development of deep coal resources is inevitable in the next several decades. As coal mining expands into deeper coal seams, the geological conditions of coal deposits exhibit notable deteriorations, such as high ground stress, high mining-induced stress, rock softening rheology and dynamic hazards. Traditional rock bolting materials and technologies is developed based on shallow burial depth and simple surrounding rock condition; however, with the increase of coal seam depth and complexity of geo-conditions, it is facing great challenges to stabilise deep underground roadway^1–6^.

Several noticeable new rock support systems have been developed to dealing with large deformation of surrounding rock in deep underground roadway, for example, large cross-section area roadway^7^, goaf-side entry under dynamic pressure^8^, soft rock roadway^9,10^ and deep roadway with complex geo-conditions^11–13^. Results showed that rock bolting could still be an effective manner to improve ground stability in deep underground roadway. Moreover, several new anchor bolts have been developed for deep underground reinforcement, such as D-bolt against roof separation^14^, He-bolt for large deformation controlling^15^ and extendable bolts for extra energy absorption^16^, and it showed that an additional rod wrapped on a certain section of the bolt could improve the cooperation of bolt with resin cartridge^17^. However, these newly designed bolts require new materials or different installation process comparing to traditional ones, leading to the increases of labour intensity and engineering cost. Therefore, it is of great value to develop new rebar bolt to improve the ground controlling effect in deep underground roadway but using current bolting equipment and process without extra engineering cost.

Rock bolting system is the primary support in underground roadway, it includes rebar bolt, surrounding rock, internal fixture (normally resin grout) and external fixture (normally plate and nut)^18^. The bonding strength between the bolt and the resin is crucial for the effectiveness of anchorage performance^19–21^. The rebar bolt profile is the point influencing the coupling effect between the bolt and the resin^22–29^. In this study, a new left hand threaded bolt is designed and tested in laboratory and in fields; its installing and bolting performances were compared to conventional left- and right-handed rebar bolts commonly used in underground coal mine under different surrounding rock conditions, which provided an experimental basis for new rebar bolt suitable for large deformational roadway support.

## Materials and methods

### New rebar bolt

**Figure 1:**
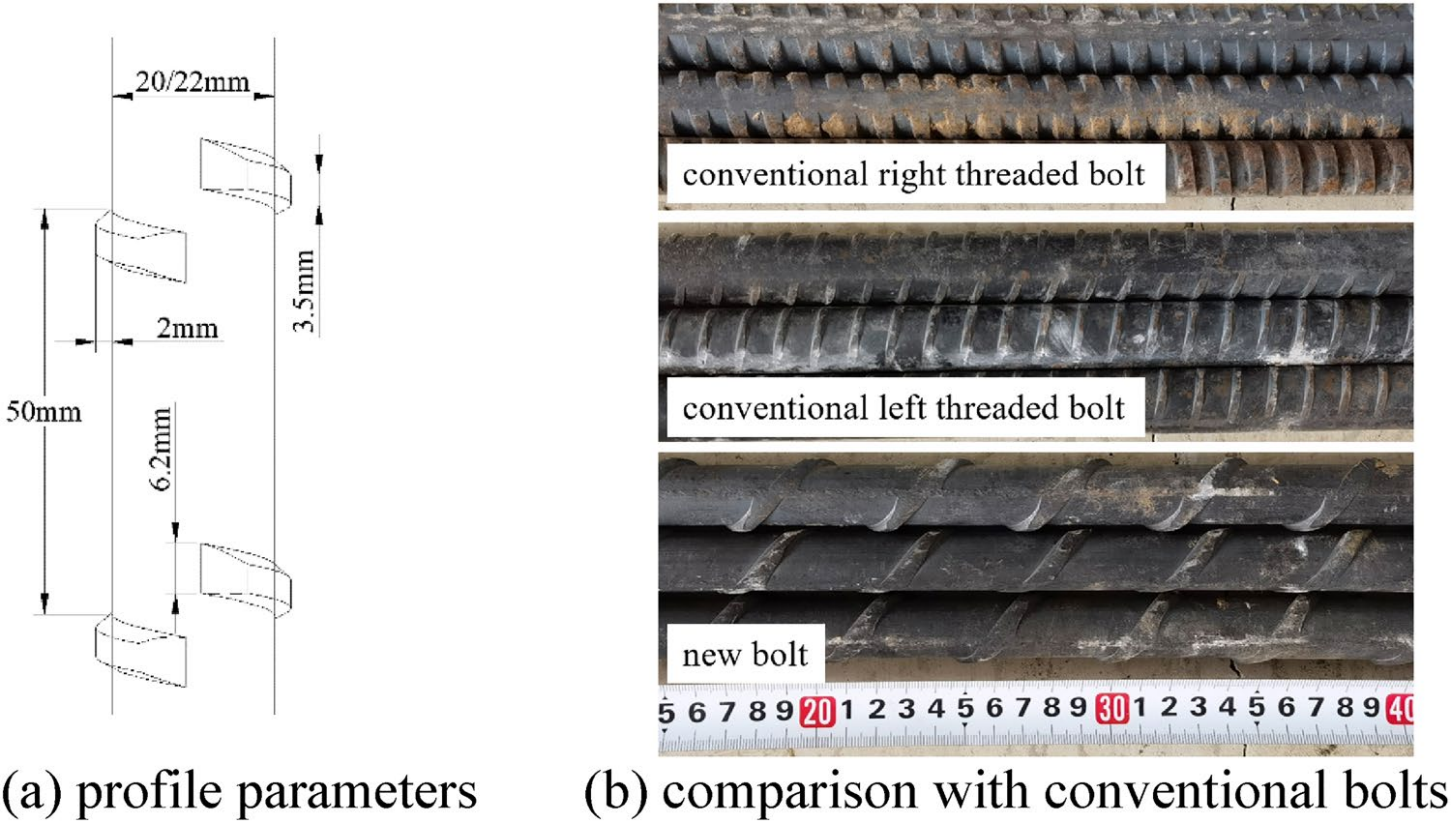
Profile parameters and comparison with conventional bolts of new rebar bolt.

The specific structural parameters of the new rebar bolt are shown in Fig. 1a. The bolt is featured by 2 mm rib height (conventional left threaded rebar is 1 mm) and 50 mm rib interval (12 mm for conventional bolts), a comparison of the new bolt with conventional bolts is shown in Fig. 1b. As shown in Fig. 1, the transverse ribs are arranged in intervals rather than in continuous spirals.

The new bolt can be manufactured using Φ18–25 mm round rod steel, with tensile strength of 335, 500 or 600 MPa. At the bottom of the hole, the bolt end is wedged; another end of the bolt is threaded. It should be noted that, under any cases, the cost of the new bolt is the same as the conventional bolt with equal diameter, as the bolt is sold based on weight. Moreover, the installation equipment and process are all same to those of conventional bolts.

### Installation test

A comparative analysis of resin mixing effect was conducted between the new bolt and conventional left and right-threaded rebar bolts commonly used in underground coal mine using a self-developed computer-controlled hydraulic servo anchorage and pullout testing machine. The resin mixing test specimens used a Ф22 mm conventional left threaded rebar bolt, a Ф20 mm right threaded rebar bolt and a Ф22 mm new bolt. The specimens had a length of 500 mm and a 30 mm inner diameter steel sleeve was used to simulate surrounding rock. The anchorage grout was CKa2333 resin (diameter 23 mm, length 330 mm), with curing time of 8 ~ 25 s, uniaxial compressive strength 43.1 MPa, Young’s modulus 19.5 GPa, Poisson’s ratio 0.26 and shear strength of 19.5 MPa.

**Figure 2:**
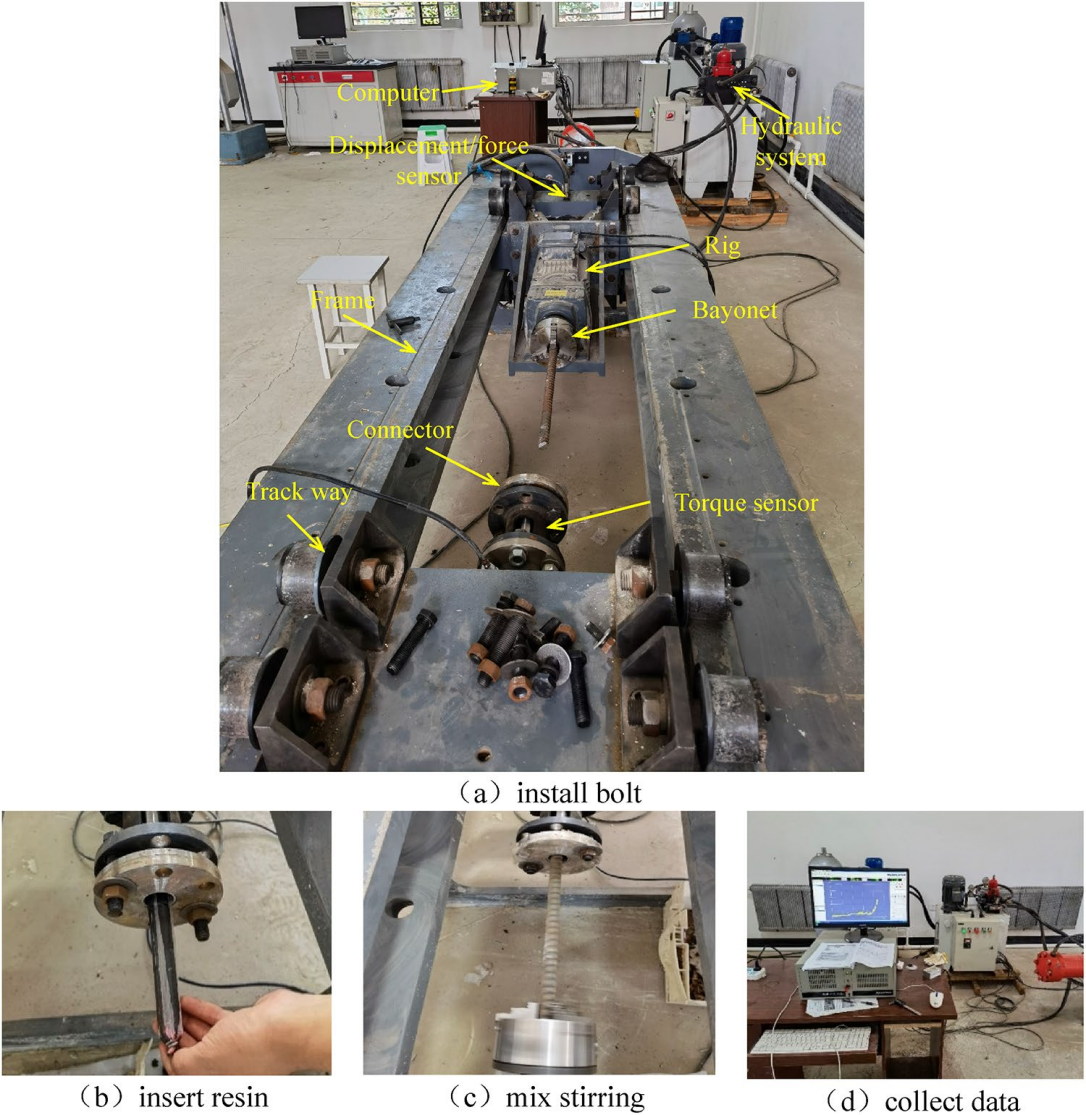
Operation flow of resin mixing test.

The testing procedure is, after switching on the testing machine, set the initial drilling speed to 250 rad/s. Clamp the bolt (no washer and nut) onto a three jaw self-centering chuck (Fig. 2a); Connect and fix the steel tube using a flange, and then insert the resin into the steel tube (Fig. 2b). Adjust the distance between the bolt and the steel tube, and start stirring (Fig. 2c). Control the drilling speed and record data in real time (Fig. 2d). Recorded data included the axial resistance force and rig torque during the bolting process.

### Laboratory pullout test

**Figure 3:**
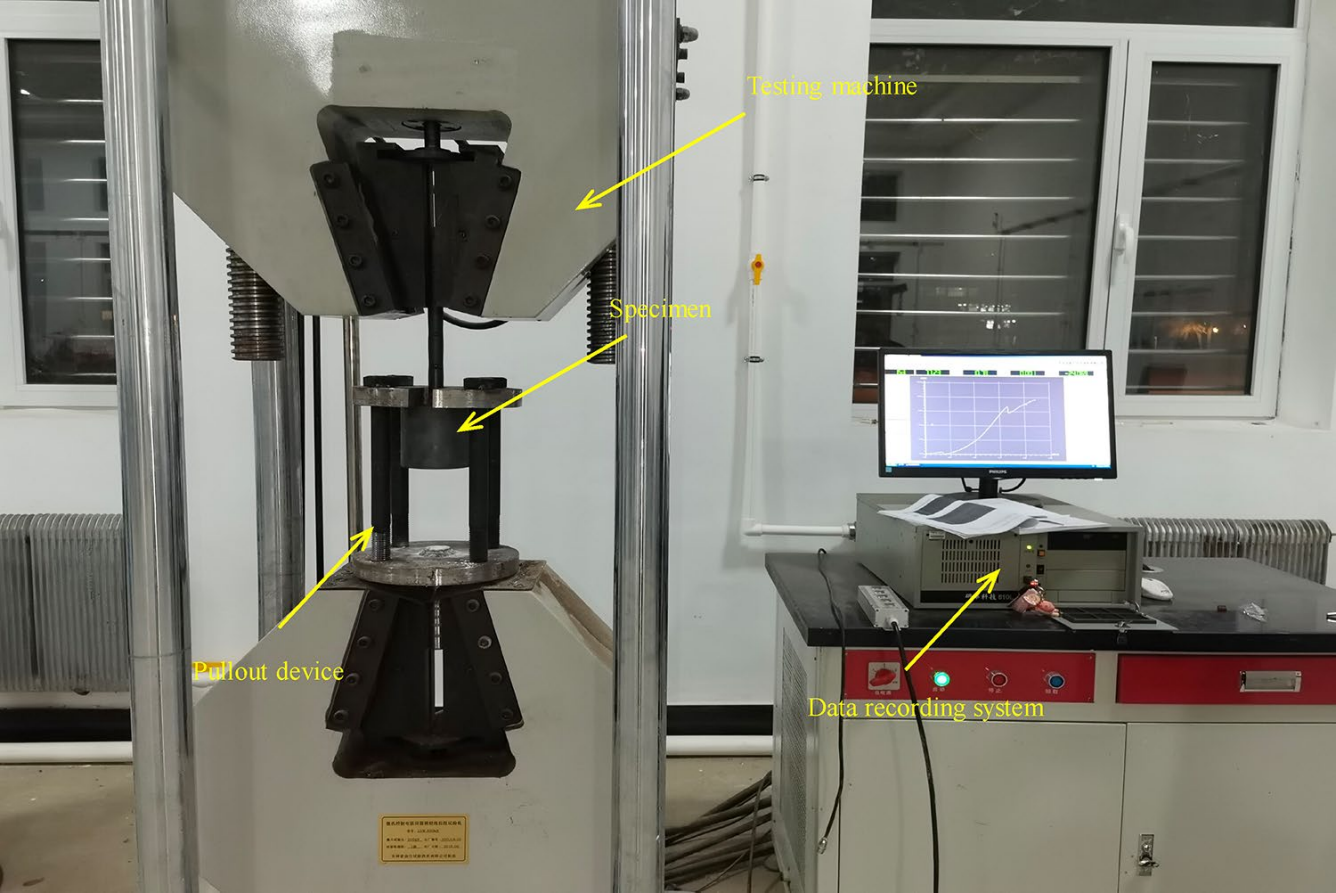
Laboratory pullout testing system.

Laboratory pullout test was conducted to evaluate the anchoring performance of the new bolt, as shown in Fig. 3. Based on previous research result^24^, PVC and aluminum sleeves were used to simulate different surrounding rock. That is, PVC sleeves with a length of 100 mm, inner diameter of 30 mm and wall thickness of 31 mm were used to simulate coal or weak surrounding rock; Aluminum sleeves with a length of 100 mm, inner diameter of 30 mm and outer diameter of 42 mm were used to simulate medium strength surrounding rock. To facilitate the production of specimens, bulk medium speed resin produced according to the MT146.1–2011 were used. The uniaxial compressive strength of the resin was measured to be 60.9 MPa, with cohesion of 19.1 MPa and internal frictional angle of 32.3°. The load and displacement of the pullout test were carried out by the electro-hydraulic servo steel strand tensile tester (JAW-500kN) controlled by microcomputer. Specific laboratory pullout tests can be referred to the literature^24^.

### Field pullout tests

To compare the bolting performance of the new with traditional bolts, field pullout tests were carried out under soft coal, hard coal and rock conditions. In each geo-condition, the bury depth of the bolt was different, there were total 35 tests were conducted, detailed information was shown in Table 1.

**Table 1:** Total testing (right-, left- and new are right-hand thread, left-hand threaded and new bolt, bury depth unit: m).

	Soft coal				Hard coal			Rock		
	0.5	1.0	1.5	2.0	0.6	1.2	1.8	0.8	1.2	2.0
Right-	2	2	2	2	–	–	–	2	2	1
Left-	–	–	–	–	2	2	1	–	–	–
New	2	2	2	1	2	2	1	1	2	2

#### Pullout test in soft coal seam

The field pullout test of the new bolt was conducted near the return air auxiliary tunnel in the 2172W working face of Qianjiaying Mining Branch, Kailuan Group. Both ribs of the tunnel in the testing area are composed of soft coal. Site inspection observed that the coal seam surface was fragmented and often peeling off, making it difficult to obtain standard coal sample for uniaxial compressive test to determine its mechanical properties.

**Figure 4:**
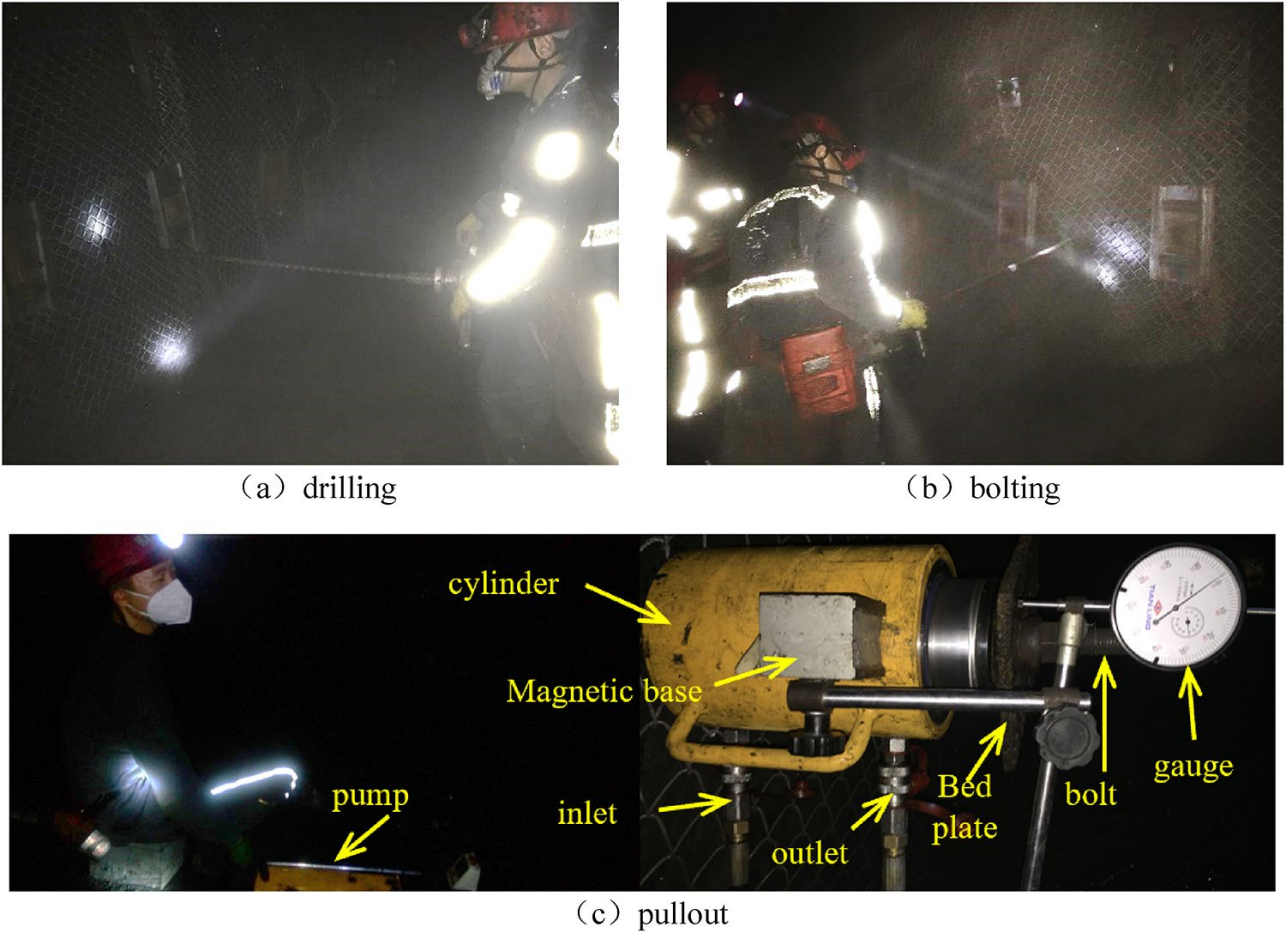
On-site bolting and pullout test.

Short pullout tests were carried out in the field. The anchorage length was designed as 300 mm. Boreholes were drilled using a 28 mm diameter drill bit at 4 different depths of 500, 1000, 1500, and 2000 mm. A conventional Φ22 mm right threaded rebar bolt was also tested for comparison. The specific information regarding the testing location, anchorage installation and pullout method followed the standard of GB/T 35056-2018, namely Technical Specifications for Anchor Support in Coal Mine Roadways^30^. On site testing procedure is shown as Fig. 4.

#### Pullout test in hard coal seam

The test was conducted in the #112201 working face of the XiaobaoDang #1 Coal Mine. The average strength of the coal was 22.4 MPa, indicating a hard coal seam. Therefore, the rib in the tailgate of the working face was selected as the test location for the anchoring performance of the newly developed left threaded rebar bolt. In the on-site pullout test, the borehole depths were designed to be 600, 1200, and 1800 mm. The anchored bolt length was designed as 300 mm. The testing method was the same as described in Pullout Test in Hard Coal Seam.

#### Pullout test in medium strength rock

The testing site selected for the experiment is the 153rd ring roadway in the northern wing of the Hengda Coal Mine. The burial depth of the testing area is approximately 790 m. The roof and ribs are both Class III medium stability surrounding rock, uniaxial compressive strength is 30–60 MPa. The original design for the anchor length in the pull-out test was 300 mm. However, the actual field data showed the pullout force reaching 600 MPa. To avoid bolt rod yielding, the anchor segment in the test was changed to 200 mm. Three different hole depths of 800, 1200 and 2000 mm were selected to compare the anchoring performance between the new left threaded rebar bolt and the conventional right threaded bolt used in the Coal Mine. The testing method was the same as described in Pullout Test in Hard Coal Seam.

## Results

### Anchorage mixing test results

**Figure 5:**
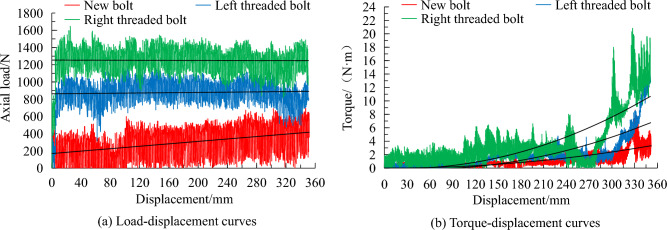
Axial resistance force and torque during bolting procedure.

Axial resistance force, torque, and displacement data were collected through sensors using the experimental equipment shown in Fig. 2a. The axial resistance force and torque during the resin mixing process of the new bolt, conventional left threaded bolt and conventional right threaded bolt are shown in Fig. 5.

The results show that the new bolt had low axial resistance force and rotation torque during the resin stirring process. At a rotation rate of 250 rad/s, the maximum axial resistance for new bolt, conventional left and right threaded bolts were 690, 1180 and 1640 N, respectively. The resistance of the new bolt reduced by 41.5% and 57.9% compared to the left and right threaded bolts, respectively. The maximum installation torques for the 3 bolts were 8.4, 14.4 and 20.8 N·m, respectively. The new bolt showed a reduction of 41.7% and 59.6% in torque compared to the left and right threaded bolts, respectively. The axial resistance and rotation torque during the bolt installation process are relevant to the labour intensity and working efficiency in the rock bolting operation. Therefore, the new bolt exhibits greater installation performance compared to the conventional bolts.

**Figure 6:**
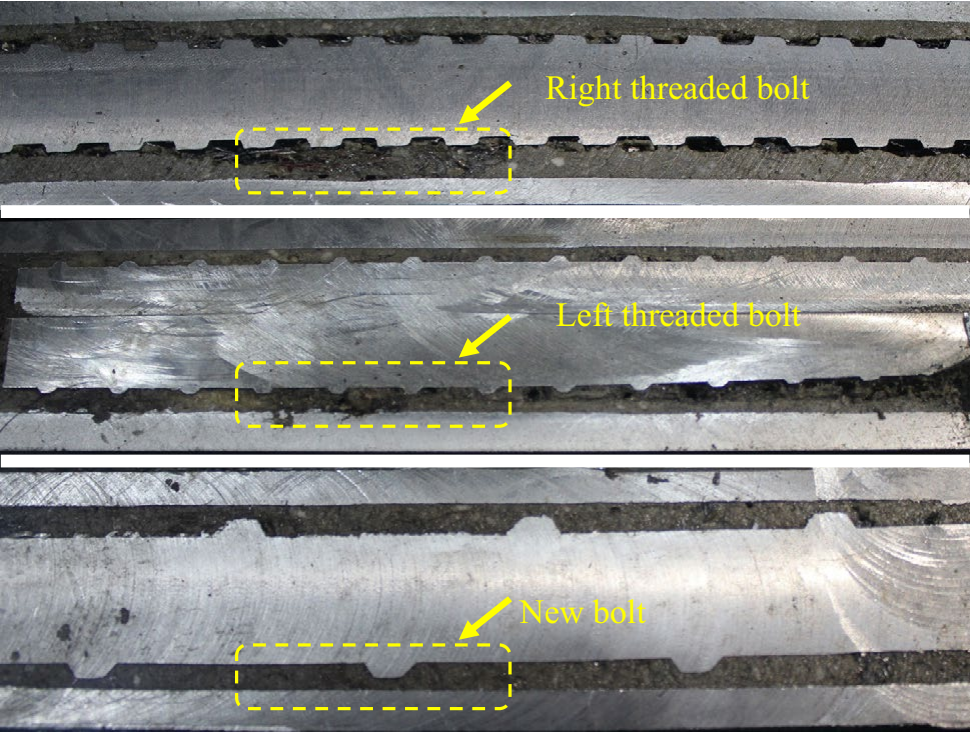
Comparison of resin filling between new bolt and conventional bolts.

After testing, the post installation specimens were sawed along an axisymmetric plane to observe their resin mixing effect, as shown in Fig. 6. It shows that there is separation between the conventional right threaded bolt and resin grout. The spacing between its transverse ribs is narrow, which makes it difficult for the grout to fulfill the groove. The grout filling effect of the conventional left threaded bolt is better than that of the right threaded bolt. For the new bolt, the resin grout fulfills the space between adjacent transverse ribs as the new bolt has large transverse rib interval, allowing sufficient space for the resin to flow during the stirring process. Therefore, the new bolt exhibits the best resin grout filling effect.

### Laboratory pullout test results

#### Pullout test result using PVC sleeve

**Figure 7:**
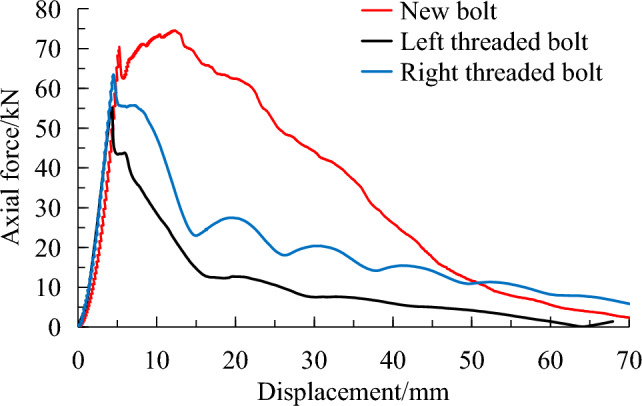
Pullout test results of new bolt and conventional bolts using PVC sleeve.

PVC sleeve is used to simulate weak surrounding rock. The typical pullout curves of different bolts are shown in Fig. 7 and the result is summarised in Table 2, among them, the energy consumption was calculated by the area enclosed by the pullout curve.

**Table 2:** Pullout test results of new bolt and conventional bolts using PVC sleeve.

Bolt	Peak axial force (kN)	Displacement at peak load (mm)	Energy absorption (J)
New bolt	76.8	11.2	2470.1
Conventional left threaded bolt	55.4	6.8	976.9
Conventional right threaded bolt	66.4	4.5	1513.1

The peak pullout force for the new bolt, conventional left and right threaded bolts are 76.8, 55.4 and 66.4 kN, respectively. This represents an increase of 38.6% and 15.7% compared to the conventional left and right threaded bolts, respectively. The displacements at the peak pullout force for 3 types bolt are 11.2, 6.8 and 4.5 mm, respectively, which corresponds to an increment of 64.7% and 148.9% compared to the conventional left and right threaded bolts. The energy absorptions of 3 kinds of anchor system are 2470, 976.9 and 1513 J, respectively, which represents an increase of 152.9% and 63.2% compared to the conventional left and right threaded bolts. In summary, under simulated weak surrounding rock condition, the new bolt shows significant improvement in axial bearing capacity, bolt displacement and energy dissipation comparing with the conventional bolts.

#### Pullout test result using aluminum sleeve

**Figure 8:**
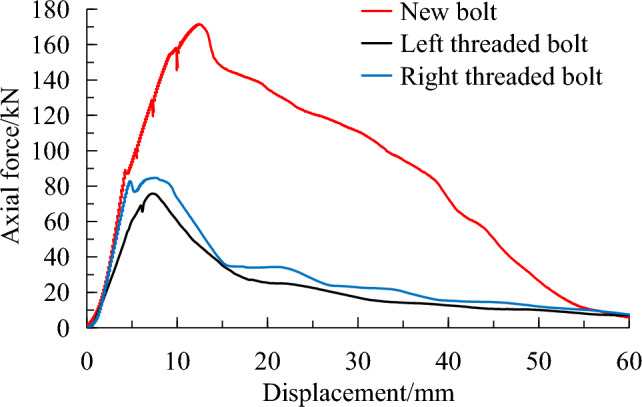
Pullout test results of new bolt and conventional bolts using Aluminum sleeve.

Aluminum sleeves were used to simulate medium strength surrounding rocks. The typical pullout curves for 3 types bolt are shown in Fig. 8, and the test results are presented in Table 3.

**Table 3:** Pullout test results of new bolt and conventional bolts using Alumimum sleeve.

Bolt	Peak axial force (kN)	Displacement at peak load (mm)	Energy absorption (J)
New bolt	180.3	15.8	5888
Conventional left threaded bolt	82.0	7.7	1901
Conventional right threaded bolt	87.3	7.4	1878

The peak pullout force of the new bolt is 180.3 kN at a displacement of 15.8 mm. Its energy absorption capacity is 5888 J, which represents an increase of 119.9%, 105.2% and 209.8% compared to the conventional left and right threaded bolts, respectively. Under the medium strength surrounding rock condition that is simulated by the Aluminum sleeve, the anchoring performance of the new bolt is significantly higher than that of the conventional bolts.

### Results of field pullout tests

#### Results in soft coal

**Figure 9:**
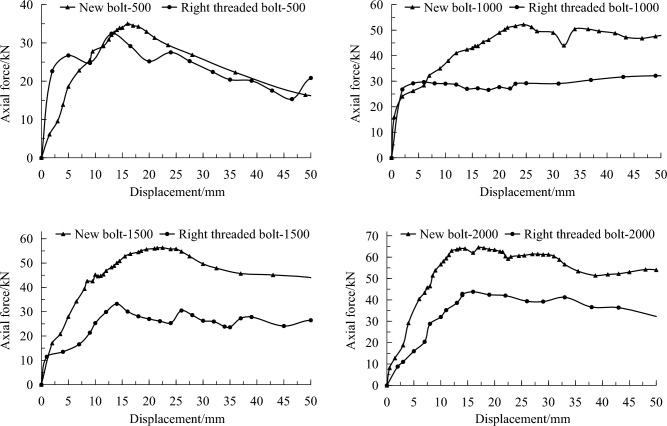
Pullout curves of soft coal at different borehole depths.

The field pullout test results in a soft coal roadway are presented in Table 4, and typical pullout curves at different borehole depths are shown in Fig. 9.

**Table 4:** Pullout test results of different borehole depths in soft coal roadway.

Depth (mm)	Peak force (kN)		Failure mode
	New bolt	Right threaded bolt	
500	33.1	29.5	Rock resin interface
1000	47.6	30.4	Resin failure
1500	57.9	34.4	Resin failure
2000	64.0	43.7	Resin failure

It shows that at a borehole depth of 500 mm, the average peak pullout forces of the new bolt and the conventional right threaded bolt are 33.1 and 29.5 kN, respectively. At a borehole depth of 1000 mm, the average peak pullout forces are 47.6 and 30.4 kN, respectively. At a borehole depth of 1500 mm, the average peak pullout forces are 57.9 and 34.4 kN, respectively. At a borehole depth of 2000 mm, the average peak pullout forces are 64.0 and 43.7 kN, respectively. Under these four borehole depth conditions, the peak pullout force of the new bolt is increased by 12.2%, 56.6%, 32.5%, and 46.5% compared to the conventional right threaded bolt, respectively. The anchoring effect of the new bolt is superior to that of the conventional right threaded bolt.

At a borehole depth of 500 mm, the rock mass fractures are developed, the failure mode of anchoring is mainly the bond failure between the resin and the surrounding rock, which is independent of the bolt surface profile. Therefore, the test data under this condition could not be used for bolting effect analysis.

#### Results in hard coal

**Figure 10:**
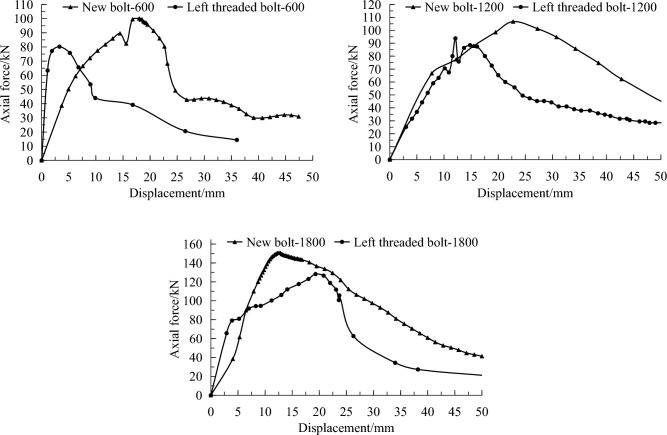
Pullout curves of hard coal roadway at different borehole depths.

Pullout tests were conducted on a hard coal roadway rib with different borehole depths of 600, 1200 and 1800 mm. The anchoring segment length was designed to be 300 mm. The comparison test results of the new left threaded bolt and the conventional left threaded bolt used in the coal mine are presented in Table 5, and their typical pullout curves are shown in Fig. 10.

**Table 5:** Pullout test results of different depths on hard coal roadway rib.

Depth (mm)	Peak force (kN)		Failure mode
	New bolt	Right threaded bolt	
600	111.4	85.5	Mainly resin failure
1200	125.4	90.7	Resin failure
1800	150.9	103.9	Resin failure

The maximum pullout forces of the new and conventional left threaded bolts at borehole depths of 600, 1200 and 1800 mm are 111.4, 125.4, 150.9 kN and 85.5, 90.7, 103.9 kN, respectively. The increase is 30.3%, 38.3% and 45.2%, respectively. It can be concluded that the anchoring effect of the new bolt is superior to that of the conventional left threaded bolt.

#### Results in medium strength rock

**Figure 11:**
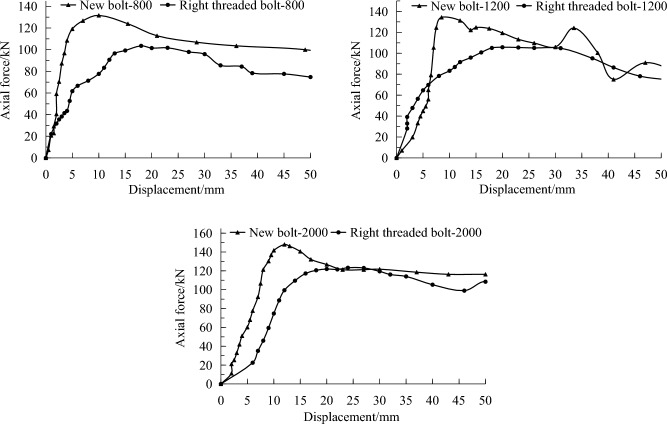
Pullout curves in rock tunnel at different borehole depths.

In the rock tunnel, pullout tests were conducted on three different borehole depths of 800, 1200 and 2000 mm, conventional right threaded bolts used in the mine were also tested for comparison. The pullout test results are shown in Table 6, and typical pullout curves are depicted in Fig. 11.

**Table 6:** Pullout test results of different borehole depths in rock tunnel.

Depth (mm)	Peak force (kN)		Failure mode
	New bolt	Right threaded bolt	
800	142.2	112.5	Resin failure
1200	148.0	115.5	
2000	162.9	125.3

At a borehole depth of 800 mm, the average peak pullout forces for the new bolt and the conventional right threaded bolt are 142.2 kN and 112.5 kN, respectively. At a borehole depth of 1200 mm, the average peak pullout forces are 148.0 kN and 115.5 kN, respectively. At a borehole depth of 2000 mm, the maximum pullout forces are 162.9 kN and 125.3 kN, respectively. Under these three borehole depth conditions, the peak pullout forces of the new bolt have increased by 26.4%, 28.1% and 30%, respectively. Therefore, it can be concluded that the anchoring effect of the new bolt is superior to the conventional right threaded bolt under medium strength surrounding rock condition.

## Discussion

**Figure 12:**
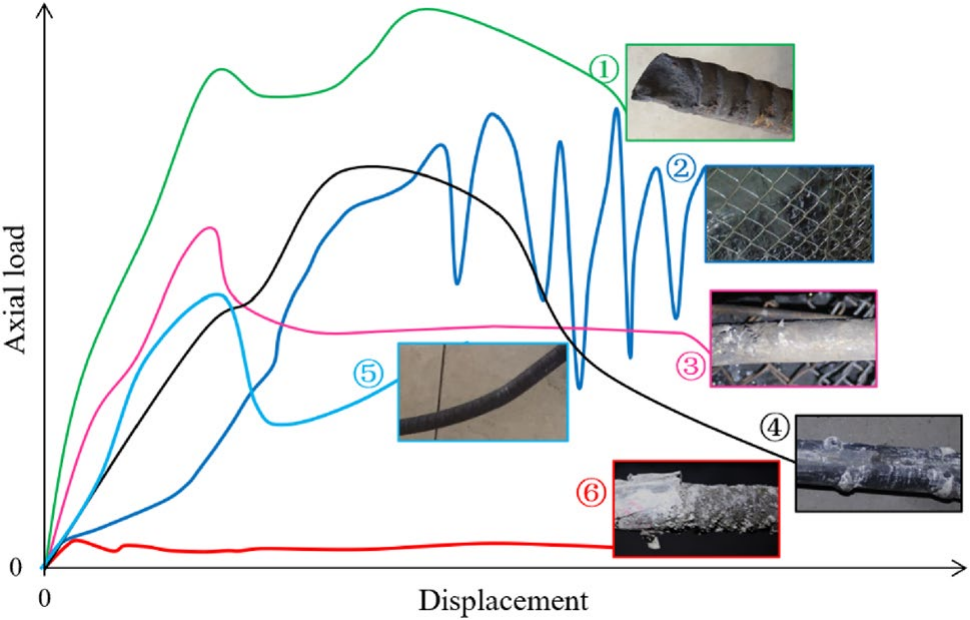
Typical field pullout curves.

Based on the pullout tests under different surrounding rock conditions, six typical pullout curves are summarized, as shown in Fig. 12.

The six typical pullout curves are as follows.

① *Rod* *fracture*: In this case, it is mainly due to the excessive length of the anchoring section or the high strength of the surrounding rock mass. Even if the pullout force exceeds the yield strength of the anchor rod, there is no large displacement or sliding with the surrounding rock. The pullout curve shows a suddenly breakage after gradually increasing the load until it exceeds the ultimate tensile strength of the rod. The shape of the pullout curve is very similar to the uniaxial tensile curve in the laboratory, and the corresponding pullout force obtained is the tensile strength of the rod.
② *Surrounding* *rock* *crushing*: As the pullout load gradually increases to a critical point, the sur-rounding rock mass near the loading end is unable to support the pullout load. The surrounding rock mass suddenly collapses with a crisp splitting sound, and the axial load drops suddenly. As the pullout test continues, the surrounding rock mass on the tunnel surface gradually compacts. When the load gradually increases to the next critical point of rock mass bearing capacity, it breaks again with splitting sound, and the pullout instrument shows a drop in load again. This situation occurs repeatedly even for several load cycles, and eventually, the bolt cannot be pulled out smoothly. This condition often occurs where cracks in the surrounding rock mass are well developed.
③ *Continuous* *friction* *of* *anchor* *grout*: After the anchoring system fails, regardless of how long the pullout displacement is, the bolt can still be pulled out at a consistent force. If the borehole depth is small, the rod can be pulled out thoroughly. It often occurs where the bolt is anchored in soft surrounding rock or there is a large amount of debris remaining in the borehole.
④ *Resin* *failure*: Bolting failure is closely relevant with the resin annulus^31^. After the bolt is loaded, the pullout force increases linearly until reaches a peak. The pullout force decreases until the bolt is smoothly pulled out. In this case, the failure mode of the anchorage is mostly the interface failure between the bolt and the resin. The obtained pullout curve can accurately reflect the anchoring performance of the bolt.
⑤ *Bending* *of* *bolt*: In this situation, the loading direction is inconsistent with the bolt axis. It occurs when the surface of the tunnel is initially uneven or becoming uneven due to high pullout load. Therefore, when selecting the testing location, it is essential to carefully survey the site and choose the appropriate location.
⑥ *Installation* *failure*: In this situation, the pullout load is very small, the bolt can even be pulled out manually. The main causes of this problem include the presence of significant cracks inside the borehole, causing the resin flowing into them during installation.

## Conclusions

1. A new left threaded bolt has been developed for deep coal mining. Bolt installation test showed that axial resistance and torque of the bolt are reduced by 41.5% and 57.9% compared to conventional left and right threaded bolts, respectively. The filling of the grouting material is satisfied.
2. Laboratory pullout test results show that when simulating weak surrounding rocks using PVC sleeve, the peak pull-out force of the new bolt is increased by 38.6% and 15.7% compared to conventional left and right threaded bolts, respectively. The displacement at the peak pull-out force is increased by 64.7% and 148.9%, and the system energy absorption is increased by 152.9% and 63.2%, respectively. When simulating semi-hard surrounding rocks with aluminum sleeve, the peak pull-out force, corresponding displacement and system energy absorption strength of the new bolt are increased by 119.9%, 105.2%, 209.8% and 106.5%, 113.5%, 213.6% compared to conventional bolts.
3. Field tests show that when anchored in soft coal conditions, the maximum pull-out force of the new bolt increases by 12.2%, 56.6%, 32.5% and 46.5% at different hole depths compared to conventional right threaded bolt. When anchored in hard coal, the new bolt increases by 30.3%, 38.3%, 45.2% at different hole depths compared to conventional left threaded bolts. When anchored in moderate strength surrounding rock, the new bolt increases by 26.4%, 28.1%, 30% at three different hole depths compared to conventional right threaded bolts.
4. Based on field pull-out tests, six types of pull-out curve morphologies corresponding to different pullout states are summarized, namely, rod fracture, surrounding rock cyclic crushing, continuous friction of grouting material, grouting material fragmentation, rod bending and installation failure.

## Data Availability

The datasets used and/or analysed during the current study available from the corresponding author on reasonable request.
